# Supplementary material for: Trends and Challenges in Plant Cryopreservation Research: A Meta-Analysis of Cryoprotective Agent Development and Research Focus
Source: Plants (Basel). 2025 Feb 3;14(3):447. doi: 10.3390/plants14030447 (PMC11821117; doi:10.3390/plants14030447)
Supplement: Supplementary file 1 [file plants-14-00447-s001.zip › Supplementary Figure.pptx]

## Slide 1
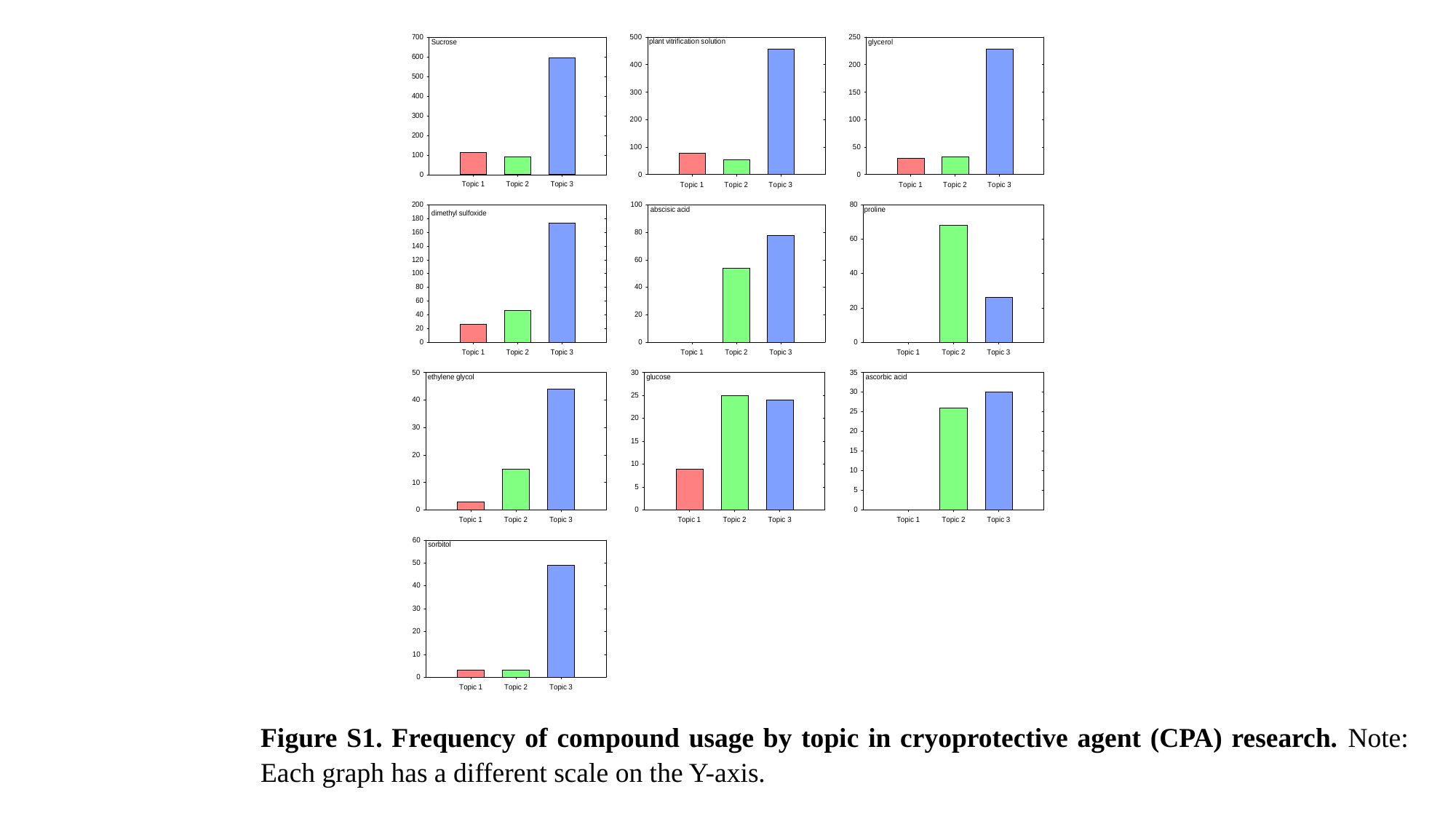

Figure S1. Frequency of compound usage by topic in cryoprotective agent (CPA) research. Note: Each graph has a different scale on the Y-axis.

## Slide 2
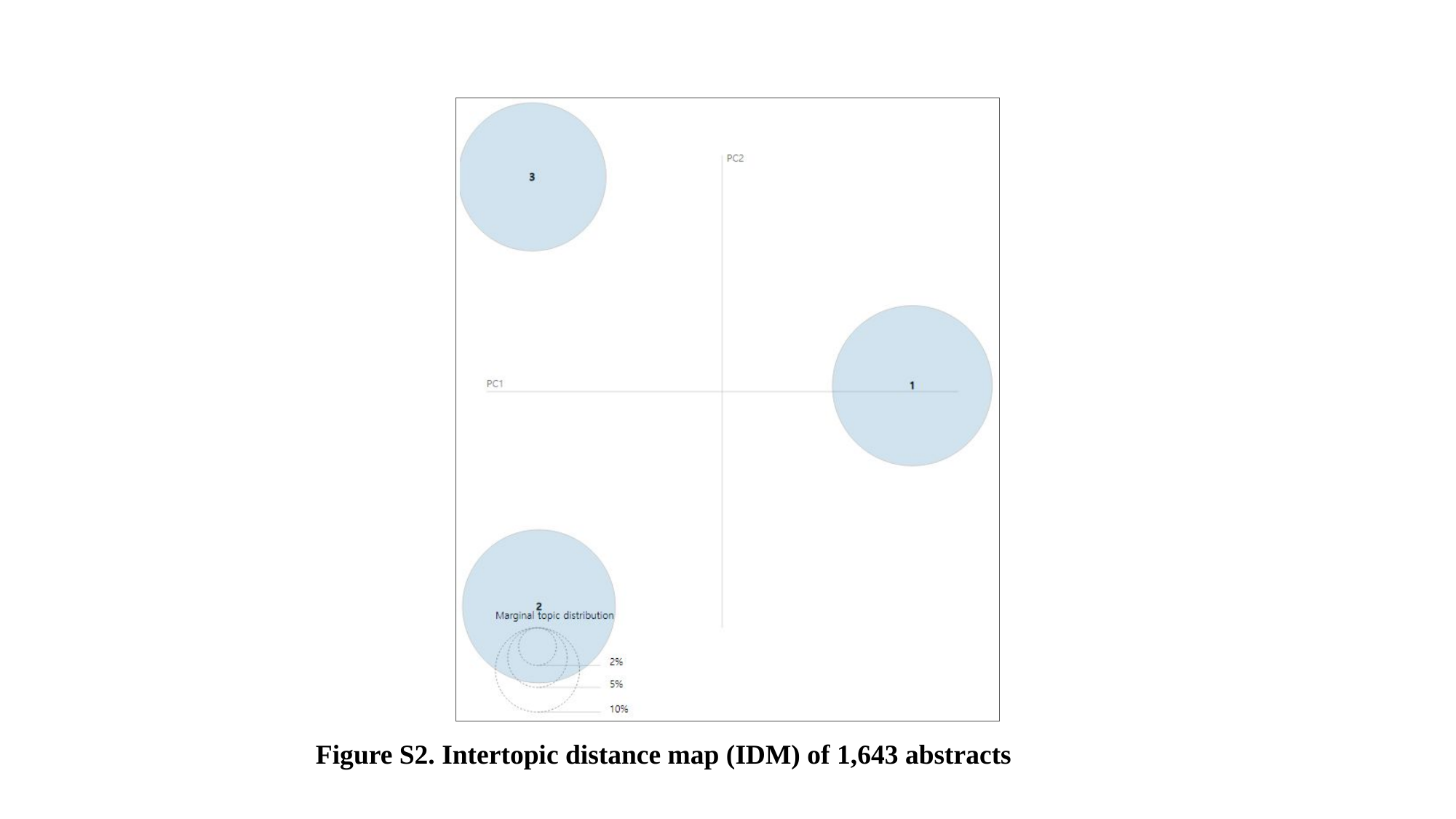

Figure S2. Intertopic distance map (IDM) of 1,643 abstracts

## Slide 3
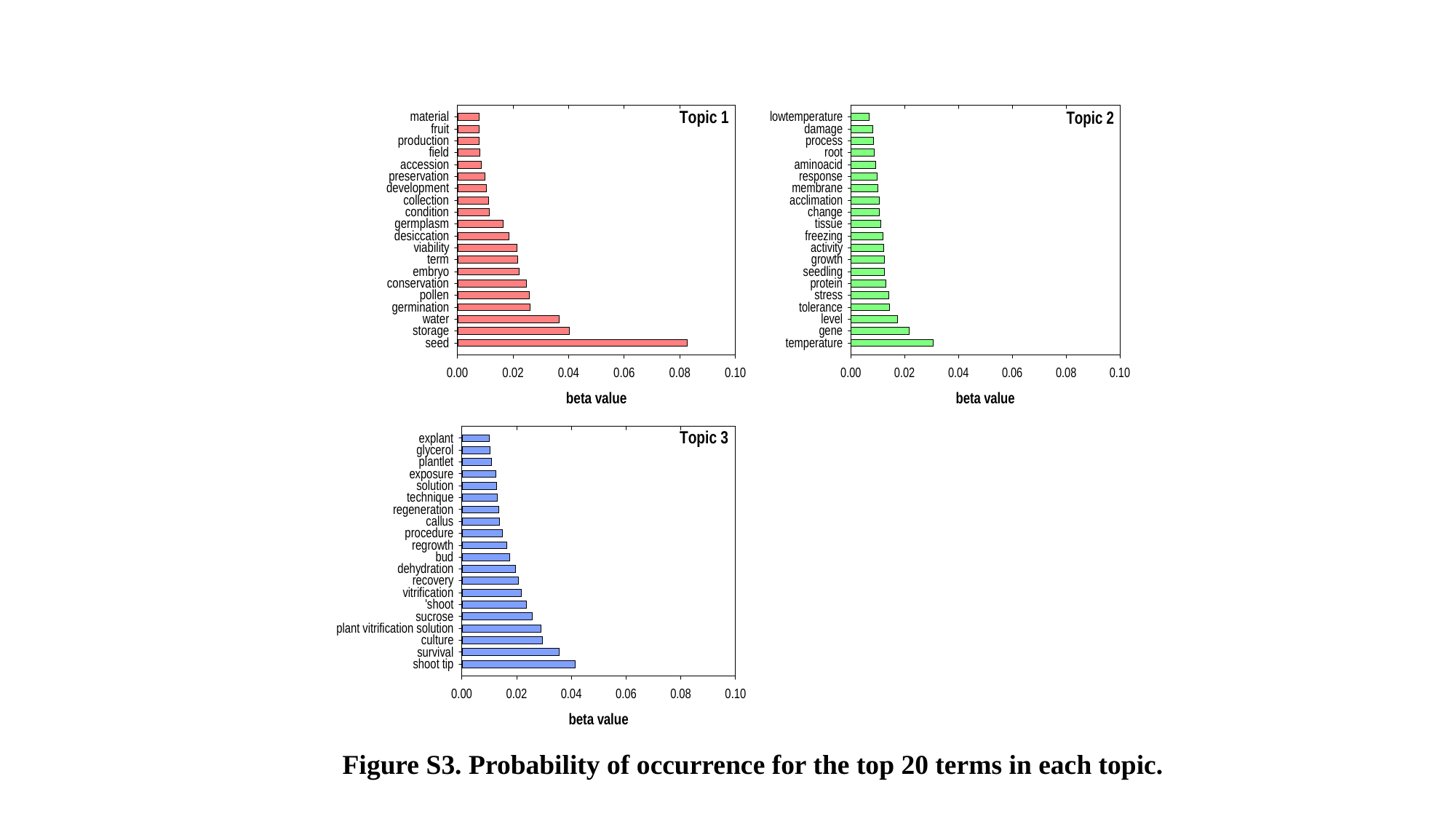

Figure S3. Probability of occurrence for the top 20 terms in each topic.
